# Two Rare Cases of Posttraumatic Peripheral Artery Pseudoaneurysm

**DOI:** 10.5334/jbr-btr.1150

**Published:** 2016-11-24

**Authors:** Stéphanie Elens, Nicolas Bossu, Pierre Puech, Johan Ghekiere, Christian Delcour, Jan Casselman

**Affiliations:** 1 Université libre de Bruxelles, BE; 2 Universitair Ziekenhuis Leuven, BE; 3 CHU de Charleroi Charleroi, BE; 4 AZ Sint-Jan Brugge, BE

**Keywords:** Pseudoaneurysm, Peripheral artery, Ultrasound-guided thrombin injection, Embolization, Digital subtraction arteriography, Angiography, Posttraumatic

## Abstract

Posttraumatic pseudoaneurysms of the lower limb are increasingly recognized due to the development of cross-sectional imaging. Two cases of anterior tibial artery pseudoaneurysm after blunt trauma are presented. The diagnostic technique of choice is Doppler ultrasound (US). In some cases, computed tomography angiography (CTA) or magnetic resonance angiography (MRA) is needed to identify the feeding vessel. The treatment of choice is not yet determined. Ultrasound-guided thrombin injection is widely used as first-line treatment, but some cases are refractory to this treatment. Further investigation and optimization of therapeutic technique to definitely exclude the pseudoaneurysm from the circulation may result in faster and more cost-effective treatment than US-guided thrombin injection.

## Introduction

A pseudoaneurysm consists of a blood collection contained by the adventitia or the surrounding soft tissues caused by dissection of all the layers of the arterial wall due to puncture, trauma, or infection [1,2]. We report two cases of a pseudoaneurysm of the anterior tibial artery revealed by persistent pain after blunt trauma and confirmed by multiple imaging modalities. First- and second-line treatments failed to induce complete luminal thrombosis or normal mobility and third-line treatment was needed. Assumed causes of this failure are discussed and appropriate time- and cost-effective diagnostic as well as therapeutic steps for the management of peripheral pseudoaneurysm are proposed.

## Case 1

**Figure 1:**
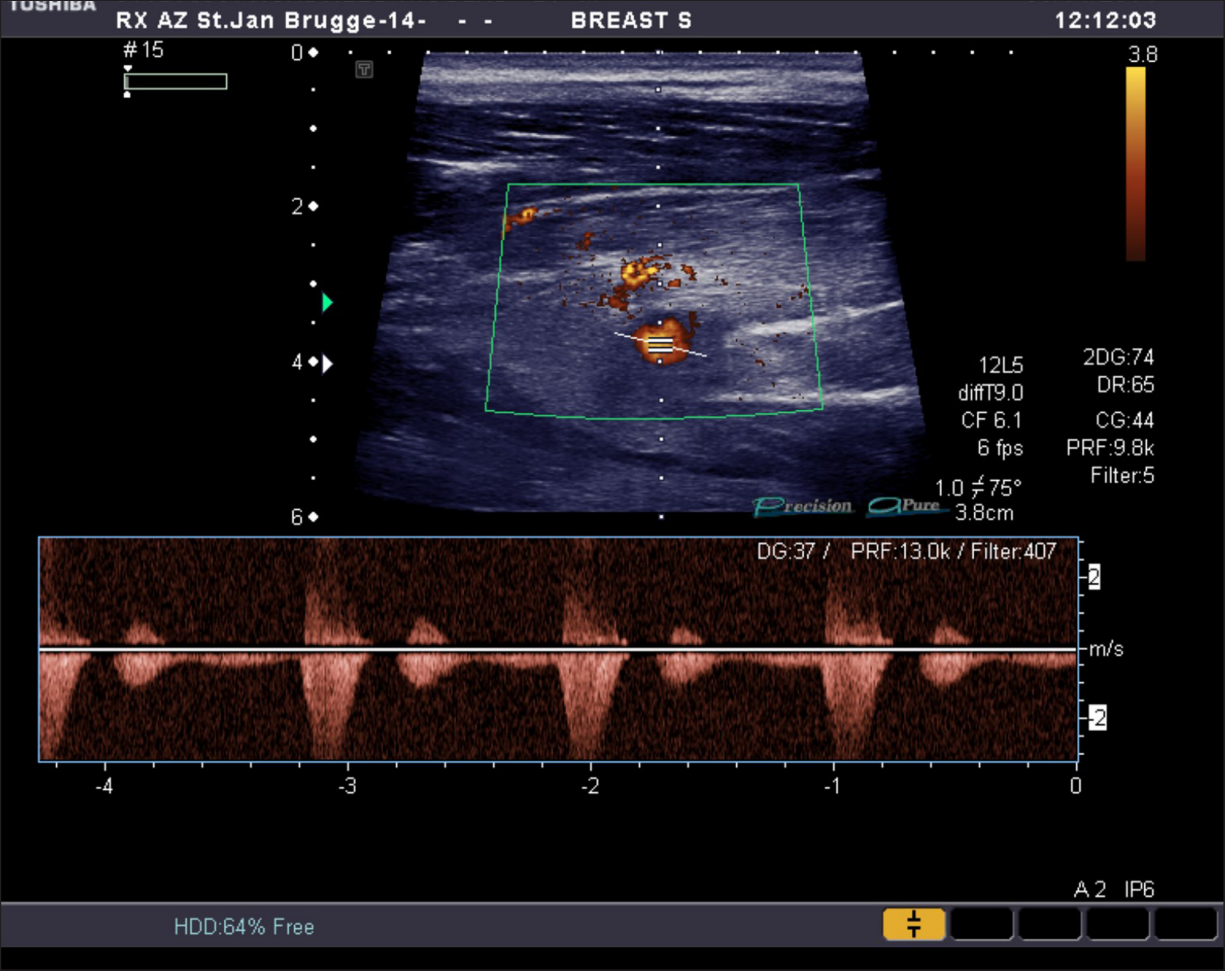
Doppler US: Arterial signal within the hematoma.

**Figure 2:**
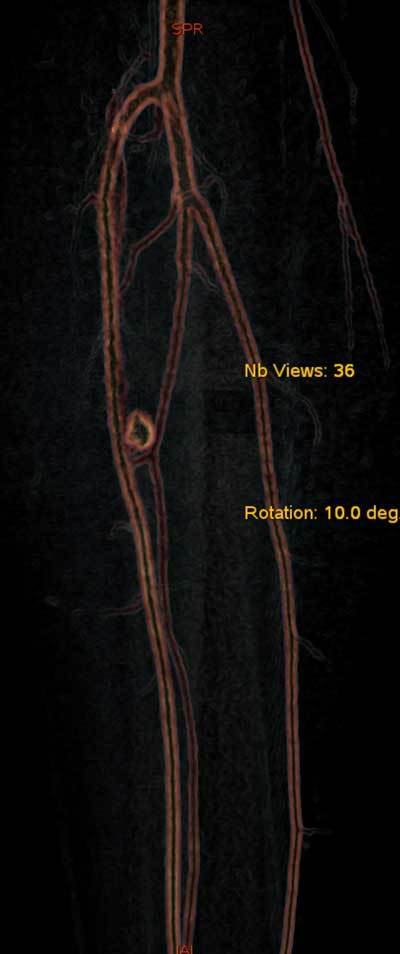
Multiplanar volume-rendering reformation CTA: pseudoaneurysm of the anterior tibial artery.

A 21-year-old professional soccer player complained of persistent pain after being tackled on his right leg. Physical examination showed tender swelling of the painful region. Plain radiographs showed no bone lesion. Ultrasound (US) examination revealed a 4 x 2 cm hematoma with an arterial pulsatile Doppler signal originating from an artery on the lateral aspect of the leg (Figure 1) suggesting the diagnosis of pseudoaneurysm of the anterior tibial artery that was confirmed by computed tomography angiography (CTA) with multiplanar reformations (MPR) of the right lower limb (Figure 2). As its greatest diameter was 6mm, the pseudoaneurysm was manually compressed under US guidance during 15 min. As no flow could be visualized thereafter, the patient was discharged with a compression bandage. Because of recurrence, thrombin (Tisseel Duo^®^, Baxter, Deerfield, Illinois) was injected within the pseudoaneurysm under US guidance. After three unsuccessful trials, the patient was referred to our tertiary hospital. A selective digital subtraction lower limb arteriography (DSA) confirmed the pseudoaneurysm of 4 mm in diameter. As discrimination between a bleeding from the main tibial artery or from one of its branches could not be made, an US-guided percutaneous puncture of the pseudoaneurysm was made with concomitant transarterial balloon occlusion of the anterior tibial artery. The correct position of the needle within the pseudoaneurysm was visually confirmed by iodinated contrast injection followed by 0.2 mL of ethylene vinyl alcohol copolymer dissolved in dimethylsulfoxide (Onyx^®^, Covidien, Dublin, Ireland). After balloon deflation, angiography showed normal enhancement of the lower limb arteries without any extravasation. The patient has recovered his professional soccer activity.

## Case 2

**Figure 3:**
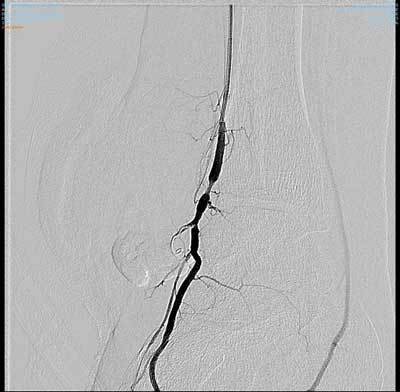
Diagnostic angiography post-embolisation. Note the coil in the feeding vessel and vasospasms. No contrast material is seen in the pseudoaneurysm.

A 43-year-old woman was treated in the emergency room for a painful ankle sprain with marked soft tissue swelling, confirmed by plain radiograph. After resolution of the soft tissue swelling, US examination was ordered for persistent pain and targeted to the periarticular ligaments revealed a partly thrombosed pseudoaneurysm but could not identify its feeding vessel. A magnetic resonance angiography (MRA) was performed and revealed that the feeding vessel was a collateral branch of the anterior tibial artery. Whilst injecting 1 mL of thrombin (Dstat^®^, Vascular Solutions, Minneapolis, Minnesota) into the pseudoaneurysm under US guidance, the arterial signal disappeared on power Doppler US. As ankle movements were still very limited, the residual hematoma was incised. Acute bleeding necessitated DSA of the lower limb that revealed extravasation of contrast material. After selective catheterization of the feeding vessel, one coil of 2 cm in length and 3 mm in diameter was deployed (MReye^®^, Cook, Bloomington, Indiana) (Figure 3) which led to the complete exclusion of the pseudoaneurysm whilst keeping the anterior tibial artery patent. Two weeks thereafter, the patient recovered complete ankle mobility and her professional activity.

## Discussion

These two cases are important to report for several reasons. First, they confirm the suggestion by Siddique et al. of pseudoaneurysm as a cause of persistent pain after lower limb trauma [3]. Second, they highlight that the first-line treatment, i.e. compression and US-guided thrombin injection, could be inefficient. Third, US examination may fail in detecting the feeding vessel, making other imaging modalities necessary.

Therapeutic approaches to pseudoaneurysm are multiple. If their size is smaller than 1 cm and the patient asymptomatic, no treatment is needed and US follow-up is sufficient [4]. Otherwise, 35 minutes US-guided compression, compression bandage, and US-guided injection of thrombin are all minimally invasive, easy to perform and inexpensive techniques [5,6,7]. Nough et al. reported that pseudoaneurysm related symptoms were due to impingement with adjacent structures and resolved with size reduction independently on spontaneous or induced thrombosis [8]. US-guided thrombin injection is currently the treatment of choice for superficial pseudoaneurysms [9]. Real-time monitoring and the possibility of multiple low dose injections make US-guided thrombin injection a safe and fast procedure [10].

As suggested by our two cases, the movements of the ankle in the immediate neighborhood of the pseudoaneurysm can lead to failure of both compression therapy and US-guided thrombin injection. Coiling under DSA guidance was indeed needed as a final therapeutic option. Various endovascular techniques including coiling and stenting have similar outcomes but coiling is preferable in distal arteries for avoiding in-stent stenosis and bending [10,11]. Finally, as associated with higher morbidity and longer hospital stay, surgery is proposed only after failure by less invasive approaches [12].

Due to the uncertainty of identifying the feeding vessel by US, multiple imaging modalities were used in these two cases but a one-step diagnostic and therapeutic angiographic approach, avoiding CTA and MRA, could be advocate in order to save time and money. This should be investigated by larger studies.

In conclusion, as a cause of persistent pain or movement limitation after lower limb trauma, pseudoaneurysm can be coiled under DSA guidance after identification of its feeding vessels during one unique procedure.

## References

[1] Mitchell RN, Kumar V, Abbas AK, Aster JC (2015). Blood vessels. Robbins and Cotran pathologic basis of disease.

[2] Anshuman D, Shekhar T, Girish C, Dwivedi SK, Ambrish K, Amit G (2006). Posttraumatic peripheral arterial pseudoaneurysms: our experience. Ind J Thorac Cardiovasc Surg.

[3] Siddique MK, Majeed S, Irfan M, Ahmad N (2014). Missed vascular injuries: presentation and outcome. J Coll Physicians Surg Pak.

[4] Mittal R, Stephen E, Keshava SN, Moses V, Agarwal S (2012). Percutaneous cyanoacrylate glue embolization for peripheral pseudoaneurysms: an alternative treatment. Indian J Surg.

[5] Pagliariccio G, Catalini R, Giantomassi L, Angelini A (2010). Management of pseudoaneurysm of the leg: is color Doppler US enough?. Journal of ultrasound.

[6] Hertz SM, Brener BJ (1997). Ultrasound-guided pseudoaneurysm compression: Efficacy after coronary stenting and angioplasty. J Vasc Surg.

[7] Cozzi DA, Morini F, Casati A, Pacilli M, Salvini V, Cozzi F (2003). Radial artery pseudoaneurysm successfully treated by compression bandage. Arch Dis Child.

[8] Nough H, Bagherinasab M, Emami M, Sarebanhassanabadi M, Hadiani L (2014). Endovascular treatment of posttraumatic pseudoaneurysms of ulnar and radial artery. Acta Medica Iranica.

[9] O’Riordan C, McCarthy E (2012). Iatrogenic lower limb pseudoaneurysms; Risk factors, diagnosis and management.

[10] Papavassiliou VG, Bell PR, Bolia A (2003). Management of a “saddle” embolus at the popliteal bifurcation by a variation of the “push and park” approach. A case report. Int Angiol.

[11] Marks JA, Hager E, Henry D, Martin ND (2011). Lower extremity vascular stenting for a posttraumatic pseudoaneurysm in a young trauma patient. J Emerg Trauma Shock.

[12] Sadat U, Naik J, Verma P (2008). Endovascular management of pseudoaneurysms following lower limb orthopedic surgery. Am J Orthop.

